# Referral of patients with emotionally unstable personality disorder for specialist psychological therapy: why, when and how?

**DOI:** 10.1192/bjb.2020.48

**Published:** 2021-02

**Authors:** Matthew Roughley, Amy Maguire, Grace Wood, Tennyson Lee

**Affiliations:** 1 East London NHS Foundation Trust, UK

**Keywords:** Borderline personality disorder, community mental health teams, personality disorders, psychosocial interventions, education and training

## Abstract

Although we commonly work with patients with emotionally unstable personality disorder (EUPD) in community mental health teams (CMHTs), only some enter evidence-based psychological therapies. Many patients are not considered ready to engage in specialist treatments and remain in CMHTs without any clear focus or structure to their treatment, which is unsatisfactory for patients, clinicians and services. We present a fictional case and synthesise available literature and lived experience to explore readiness and ways to promote it. We highlight relevant issues for trainees to consider in practice. Patients with EUPD who have not received specialist treatment can be considered in terms of the transtheoretical model's stages of change. Identifying a patient's stage can help guide how to increase readiness for referral and decide when to refer. Interventions available to all healthcare professionals which may promote readiness include: psychoeducation, personal formulations, crisis planning, goal-setting, peer support, distress tolerance skills, motivational interviewing and mindfulness.

## Case scenario

You are a psychiatry specialty trainee in a community mental health team (CMHT) seeing Miss ML in a routine appointment. She is a 29-year-old woman with emotionally unstable personality disorder (EUPD). She presents repeatedly with suicidal thoughts and has had numerous psychiatric admissions. She uses cannabis and alcohol in a binge pattern. She self-harms regularly, typically following difficult interpersonal events. You often feel unsure how you are helping Miss ML other than monitoring her mental state and risk and reviewing medication. At times you have felt irritated with her; she expects help but does not seem to help herself.

In the appointment, Miss ML requests therapy. She says she wants help with all the bad feelings, and wants to stop cutting but does not know how. She asks you why she feels like this, and what does personality disorder even mean? Miss ML complains that nobody understands her or is helping her. She asks you directly: ‘Are you going to refer me for therapy?’ You feel anxious as an earlier referral was declined by the personality disorder service. You feel an enormous pressure to say you will refer her again. What will you do?

In this paper we consider the following questions:

• *Why* am I referring for psychological therapy?
• *When* should I refer for psychological therapy?
• *How* can I help a patient reach a stage where they are ready to engage in psychological therapy?

## Introduction

We commonly work with patients with EUPD in secondary care mental health services. The prevalence of EUPD in this setting is estimated at 20%,^1^ and it is associated with considerable suffering, psychosocial impairment and high resource use.^2^ A number of evidence-based treatments are available, developed from cognitive or behavioural therapies (e.g. dialectical behaviour therapy, schema-focused therapy) or from psychodynamic/psychoanalytic origins (e.g. mentalisation-based treatment, transference-focused psychotherapy).^3^ A significant number of patients, however, will never enter such treatment. There are many different reasons for this, but a proportion of patients do not reach a stage where they are considered ‘ready’ to enter treatment. This potentially represents a lost opportunity to improve functioning and reduce suffering.

Patients with EUPD who are not ready for specialist treatment may remain in CMHTs, which poses recognised difficulties. Chaotic and risky behaviour and social problems often interfere with treatment. Resolving these ‘exclusion criteria’ can seem unrealistic.^4^ Professionals report feeling isolated and inexpert and that CMHTs are an interim ‘no man's land’, where referrals for specialist treatment are difficult. Patients report experiences of not being helped and being passed around services (Box 1).^4–6^

When considering psychological treatment for EUPD, the National Institute for Health and Care Excellence (NICE) advises considering the patient's choice and preference, willingness to engage, motivation for change, ability to work within a therapeutic relationship and availability of support. However, they make no recommendations regarding how to increase a patient's readiness for psychological therapy.^7^

A systematic search was conducted to identify literature addressing enhancing readiness of patients with EUPD for therapy. The EMBASE, PsycINFO and CINAHL databases were searched using the National Health Service's Healthcare Databases Advanced Search from inception to January 2019, combining terms relevant to personality disorder (personality disorder*, EUPD), therapy (therap*, treat*) and readiness (readiness, prepar*). None of the studies identified specifically examined methods to increase readiness in EUPD. A theoretical model suggesting factors influencing treatment readiness in personality disorder was identified. Internal (patient) factors included: cognitive (problem recognition, belief in ability to change), affective (emotional states and regulation), volitional (motivation and pursuit of goals), traits (impulsivity), relating (ability to trust and form a therapeutic alliance) and comorbidity (co-occurring psychiatric or medical illnesses). External factors included those related to the patient (current life stressors, support network and practical barriers to attendance) and service factors (accessibility, availability, staff skill and motivation).^8^

## Practical management

### Why am I referring?

Clinicians should be mindful of why they are referring *this* patient at *this* time? Although specialist EUPD treatments have demonstrated effectiveness, referrals which are declined or do not lead to treatment may lead to patients developing negative views about services and damage confidence in their ability to change.

Indicators that a patient is not currently suitable for psychotherapy include: gross instability of accommodation or finances, marked chaotic or risky behaviour, and harmful or dependent alcohol or drug use.

If considering referral despite such factors, the clinician should regard their own countertransference and whether they are referring as a defence against feelings of anxiety, despair or even countertransference hate (see below). It may be more appropriate to acknowledge their own and the patient's feelings and construct a plan to work towards referral using the interventions suggested below.

Box 1 Patient perspective.

‘It felt unjust, unfair and I was mystified when I was told I was not ready. The consultant spoke to me like a child. They explained it was better to wait than fail trying, which I appreciate more now but I was furious at the time.

‘The main issue affecting my readiness was alcohol. I had tried and failed to stop drinking for years. Alcohol was my coping mechanism and they wanted to me to stop but without giving me other ways to cope. No service knew what to do with me and I was passed around. Eventually I found Alcoholics Anonymous who really helped, they tolerated my erratic behaviour and through them I met a community of other people with lived experience of alcohol misuse and some with personality disorder. A homeless charity provided practical support. Eventually my CMHT consultant arranged a joint meeting with the alcohol service and the personality disorder service to try and find a way forward. This felt like a special gesture and that they were serious about helping me. I wonder if I had sometimes been testing teams to see if they cared.

‘Becoming ready for therapy took years and was like chipping away at a rock. I attempted suicide four times. Maybe if joint meetings and developing clear plans had occurred sooner I would have been passed around less and my journey would have been quicker and smoother.’

Patients may be displeased at a suggestion that they are ‘not ready’, and this this should be communicated with care and validation. Senior team member support may be beneficial. Box 2 contains an example of how this could be discussed with a patient.

Box 2 Communication suggestions for clinicians about readiness.

‘It is really positive you have asked for help with [list problems]. This is an important first step. To take things forward from here and to benefit from a specialist therapy, you would first need to address [specify issues]. The reasons for this are, e.g.

• Therapy focuses on how you think and feel. For therapy to work, you need to be in touch with how you are thinking and feeling. [Alcohol/illicit substances] can block or numb your thoughts and feelings, which although it can help in the short-term, will stop therapy working. We want to work with you, but you will need to reduce [alcohol/illicit substances] and we can support you through this.
• Therapy can at times make people feel very distressed and uncomfortable. At the moment, owing to [self-harm/active suicidality], we do not think it would be safe for you to start therapy as it could increase this. We will think with you about your crisis plan and ways to help you reduce [self-harm/suicidal thoughts].

I realise what I say may be frustrating, but there are things other than therapy that can help such as [suggest interventions], which may also help us in working towards a referral for therapy.’

### When should I refer?

Readiness for referral can be considered in terms of the transtheoretical model of stages of change;^9,10^ in particular, the *precontemplation*, *contemplation* and *preparation* stages.

Patients in the precontemplation stage are not aware of having a problem, and there is no current intention to change behaviour. They would be unlikely to recognise a diagnosis of personality disorder or any contribution of personality traits to their problems. They do not see a requirement to change their behaviour or have psychological therapy. Some patients may report a wish to change in response to external pressure, e.g. from family or social services. Once external pressure is reduced, engagement may dwindle.

Patients in the contemplation stage are aware that a problem exists and are seriously thinking about overcoming it but have not made a commitment to take action. They have awareness of difficulties relating to personality traits and express wishes to address these and change their behaviour. They are considering the benefits of change in comparison with the energy and effort of change.

Patients in the preparation stage combine intention and some behavioural change. They have made some reductions in problem behaviours but have not yet taken effective action, although they intend to do so soon. They would be likely to recognise a diagnosis of personality disorder, have the intention to change and be making small behavioural changes, e.g. reducing self-harm or substance misuse. We suggest that patients in this stage are most appropriate for referral.

The transtheoretical model has previously been applied to EUPD by Livesley in his integrated treatment model.^11^ This highly developed framework for treating personality disorder combines and coordinates different treatment modalities. Our simpler pragmatic approach is aimed at generalists and is not a specialist treatment. Only one study has examined the stages of change in relation to EUPD; it showed that patients in precontemplation were most likely to drop out of specialist treatment.^12^

Readiness for referral is also related to the services available, which may have differing referral criteria and work with patients at different stages of readiness. Liaison with the local personality disorder service (see below) will help clarify this and determine the likelihood of successful referral.

### How can I help a patient with EUPD become ready to engage in psychological therapy?

The transtheoretical model also describes the processes by which change occurs.^9,10^ The processes of change that are important in the precontemplation and contemplation stages include: consciousness raising (increasing awareness of the causes and consequences of their problems), self re-evaluation (assessment of self-image with and without problem behaviours) and environmental re-evaluation (assessment of how behaviour affects their environment, including relationships). Processes that are important at later stages but which seem significant in EUPD include: self-liberation (belief that change is possible and commitment to act), contingency management (consequences of taking steps in a particular direction), counterconditioning (learning healthier behaviours to substitute problem behaviours) and stimulus control (avoiding triggers of behaviour).

Many commonly used interventions (see below) utilise one or more of these processes, which may help patients to progress from one stage to the next. They can be used by any healthcare professional when trying to enhance readiness for referral. The choice of intervention will be guided by the patient's current stage of change, preference and available resources. We suggest focusing on one intervention at a time to avoid care becoming confused.

### General principles

Some strategies and clinical issues are relevant at any stage of readiness.

#### Common service factors

Successful treatments for EUPD (specialist or generalist) have common factors including:

• Focus on the therapeutic relationship, empathy and validation;
• promotion of patient self-agency;
• helping patients identify their emotions and the connections between events, emotions and behaviours;
• clinicians observant of their own thoughts and feelings and an active system for support and supervision.^13^

Structured clinical management is a manualised generalist approach utilising these factors and may be effective.^13^

#### Transference and countertransference

Clinicians should be aware of their thoughts and feelings towards patients with EUPD and how these may influence interactions and decision-making. Thoughts and feelings commonly evoked by these patients include: anxiety, rescue fantasies, anger, guilt, failure and even hate.^14,15^ If not processed, clinician responses can be unhelpful or even dangerous; for instance, malice, when the clinician may be sadistic or cruel, and aversion, which tempts the clinician to abandon the patient.^15^ These dynamics can also be played out at a systems level and affect whole teams or services.

Managing countertransference is vital to accepting, tolerating and containing such feelings. A sudden decision to refer or discharge a patient needs to be assessed for whether it is an acting out of the countertransference. Although this is a complex field, one approach is for the clinician to first recognise their thoughts and feelings, digest and try to understand them, then consider their response to them and whether this seems appropriate or not.^16,17^ Supervision or Balint groups can be used to explore transference and countertransference reactions.

#### Trauma

Patients with EUPD may have experienced trauma and during the assessment phase should be sensitively asked whether they wish to disclose trauma. Trauma-informed approaches advocate thinking ‘what happened to you?’ as opposed to ‘what is wrong with you?’

A number of principles of trauma-informed care overlap with the general principles discussed above. These include trusting and transparent relationships between clinicians and patients, collaboration, patient empowerment and choice. Clinicians should also be mindful of the risk of inadvertent re-traumatisation in their interactions with patients.^18^

Some patients may agree with a formulation describing how traumatic experiences might influence interpersonal problems, and may meet caseness for EUPD but disagree with a ‘personality disorder’ diagnosis. In this situation, the authors suggest trying to ascertain which problems and goals are a priority for the patient to address, with further discussions delegated to specialist personality disorder and trauma services to determine which therapeutic approach may be appropriate initially. There is debate regarding the overlap of personality disorder and complex trauma, but this is beyond the scope of this article.

The possibility of active trauma, e.g. domestic violence, should also be considered, both for patient safety and as it would impair readiness. Clinicians can provide advice, support and signposting to relevant organisations and consider whether safeguarding is indicated.

#### Specialist personality disorder services

If a patient is not ready to engage in specialist treatment, personality disorder services should provide advice and support to CMHTs. This can include linking a personality disorder service team member to each CMHT. This liaison service can help by discussing referrals, advising on interventions and providing feedback if referrals have been declined or treatment not initiated. Joint meetings and shared planning on how to increase readiness should be offered. Some services use a shared active list of patients in the pre-treatment stage as a means of supporting and sharing responsibility with CMHT members. In addition, personality disorder services should develop and provide training locally.^7^

#### Continuity

Therapeutic alliance and relational continuity are of particular importance when working with patients with EUPD; change of team members can be experienced as a re-enactment of loss or abandonment and thus should be avoided where possible.^13,19^ However, this is challenging in CMHTs with turnover of staff and trainees.

Although junior doctors change rotation it is essential for their training to gain experience in assessing and managing patients with EUPD. The transition between trainees should be recognised as potentially difficult and planned for with clear communication and structure. Personal formulations, crisis plans and goals should be handed over to aid continuity.

NICE provides little guidance on the role of care coordinators in EUPD.^7^ More broadly, the Care Programme Approach is indicated for patients who are at high risk and require multi-agency support, active engagement, intense intervention and support with dual diagnoses.^20^ Whether patients meet this threshold is decided on a case-by-case basis. A recent Royal College of Psychiatrists position statement recommends that all patients in Tier 2 services (and above) be allocated a long-term lead clinician who can support the patient through the engagement process.^19^ In our experience, patients with EUPD present with a very wide range of functioning, risk and support needs, and we suggest that care coordination is decided on a case-by-case basis. Most CMHTs would require a significant increase in the number of care coordinators to facilitate meaningful input for all patients with EUPD. Possible alternatives include use of support workers and peer support workers, with appropriate supervision, as a source of continuity and assistance with goals. However, if more than one clinician is working with a patient, clear communication and coordination are essential to avoid splitting or a confusing approach.

#### Validation

Patients with EUPD may have experienced invalidating environments.^21,22^ Validation and the process of listening and understanding is central to many therapies for EUPD.^23,24^ Levels of validation include: being attentive and alert, enquiring then reflecting back the patient's reported thoughts and feelings, reflecting back observed non-verbal communication, and validating the patient's experience based on the current context and their personal history.^24^

### Interventions suggested for patients in precontemplation stage

#### Psychoeducation

Educating patients (and significant others) about EUPD is an intervention in itself.^25^ The diagnostic criteria, e.g. difficulty with relationships, emotion dysregulation, impulsivity and hypersensitivity, can be linked to examples offered by the patient.^13^ Giving the diagnosis can be used to stimulate reflection. Information can also be provided about the range and nature of treatments available.

#### Personal formulation

Providing a diagnosis alone is insufficient; co-constructing a personal formulation is key in exploring a person's understanding of their problems.^19^ One approach is the ‘5 Ps’ model (problems, predisposing, precipitating, perpetuating and protective factors). Through this process, ways to avoid or challenge precipitating and perpetuating factors and strengthen protective factors can be identified, as can goals to work towards.^17^

#### Goal-setting

Clarifying a patient's goals, identifying obstacles to goal attainment and considering how therapy might assist with these may increase motivation to enter treatment.^26^ Encouraging a patient to evaluate how they and their life may look different in relation to their goals could be part of this. Goals should be specific, with defined patient and professional responsibilities. The clinician can help identify manageable short-term treatment aims with achievable steps. Long-term goals, e.g. those relating to employment, can give direction to the treatment strategy.^7^

#### Crisis planning

Collaborative crisis planning is important as part of risk management and can be seen as an early form of treatment contracting. It promotes safety and quicker recovery from crises. Steps include identifying triggers, thoughts and feelings associated with an emerging crisis, actions that can avert an escalating crisis and actions to avoid when in crisis.^13^

#### Substance misuse

Clinicians should assess the level of misuse regularly and clarify its function. Active substance misuse reduces the benefits of therapy, and harmful or dependent users are unlikely to be accepted into specialist treatments. NICE advise referring patients with EUPD and dependence on alcohol or substances to appropriate services; the care coordinator should remain involved and provide information on community support networks, e.g. Alcoholics Anonymous.^7^ Distinctions can be made between patients using as a form of self-harm, using to manage emotions, and dependent use, although overlap does occur. If the use is viewed as self-harm, general strategies to reduce self-harm can be applied, such as delaying use after an urge, distraction, relaxation or finding other outlets. Chain analysis can explore and link events leading to use.^13^

#### Distress tolerance skills

These skills help patients to manage intense emotional states, recognise triggers and endure negative emotions so that problem-solving can occur. Distress tolerance skills include distraction, self-soothing, relaxation and acceptance.^27,28^

#### Housing and finances

Stressors such as housing and finances may affect readiness for treatment.^8^ Support in stabilising a patient's social situation is a therapeutic intervention and may support building a therapeutic alliance.^13^ Maslow's hierarchy of needs could be used as a visual psychoeducational tool to explain the importance of addressing physiological and safety needs before focusing on ‘higher’ needs.^29^

### Interventions suggested for patients in contemplation stage

#### Motivational interviewing

Fluctuating engagement may be related to ambivalence about change.^30^ Using an overly directing style with patients can result in resistance or passivity. Motivational interviewing involves helping patients to say what they want to change, identify why (pros and cons of change), gain confidence in their ability to change and consider how they might change.^31^ Motivational-based interventions can increase motivation and confidence, and decrease substance use and risky behaviours.^26,32^

#### Mindfulness

Mindfulness emphasises being present in the moment and increases awareness and acceptance of experiences, which fosters emotional processing and distress tolerance.^33^ Mindfulness offers insight into the ‘process’ of specialist treatments, as it creates a space between thoughts and feelings.^34^ Improvement in attention and impulsivity was demonstrated when mindfulness was practised alongside general psychiatric care in patients with EUPD.^35^

#### Peer support groups

Learning from other patients at different stages of change can provide patients with evidence that change is possible. Service user network (SUN) projects are community-based support groups for patients with EUPD. They can help patients develop ways of coping and reduce crises. Use of SUN projects is associated with improved functioning and reduced use of services.^36^

#### Offering a range of interventions

Specialist treatments are a significant commitment for patients in terms of time and emotional expenditure. Initial use of lower-intensity or alternative therapies, e.g. art, music or movement, could provide an introduction to the nature of therapy, attending to a frame and developing a trusting relationship with a therapist. Therapies which are not based on verbal communication may also be more acceptable to some patients. Although these are not evidence-based treatments for EUPD, their completion could lead to referral for specialist treatment.

#### Volunteering or employment

Activities that help create structure and promote responsibility and confidence in ability to change may be beneficial. Some CMHTs have access to employment advisers with experience working with people with mental health problems, who can be particularly helpful.

Table 1 groups the interventions suggested above into internal and external factors and according to the stage of change of the patient.

**Table 1:** Interventions which may increase readiness for referral for specialist treatment

Factors affecting readiness	Stage of change	
	Precontemplation	Contemplation
Internal	• Psychoeducation regarding personality and diagnosis • Personal formulation • Collaborative goal-setting • Crisis planning • Distress tolerance skills • Address substance misuse	• Psychoeducation regarding therapy • Collaborative goal-setting • Mindfulness • Motivational interviewing • Lower-intensity or alternative therapies, e.g. art, music or movement therapy • Peer support groups, e.g. SUN project
External	• Key worker or peer support worker • Support for dependents or carers • Support or signpost regarding domestic violence or other safety issues • Support or signpost regarding social stressors, e.g. accommodation, finances	• Support with employment or volunteering • Address practical barriers to attendance, e.g. bus pass • Support or signpost regarding social stressors, e.g. accommodation, finances • Liaison with personality disorder service

### Should I discharge?

**‘**Precontemplation’ suggests that change will be considered at some time in the future. Experience suggests this does not always occur, raising the issue of how to manage such patients. To the best of our knowledge, no evidence exists regarding whether to continue to try to engage patients in a CMHT or discharge them. Opinions and practices vary.

We suggest that patients in precontemplation should be offered interventions as above, with an agreement between patient and clinician regarding timeframe and responsibilities, e.g. attending appointments, setting goals, and following crisis plans. After the agreed timeframe, if there has been no clear benefit or effort to work towards goals or adhere to responsibilities, then discharge could be considered. NICE advises discussing the discharge process with the patient and agreeing a care plan with steps to manage distress, cope with future crises and re-engage in the future.^7^ This should be clearly communicated to the general practitioner, including how they can access support.

However, this approach may be challenging for patients with repeated risky behaviour. It may be more pragmatic not to discharge but to focus on promoting safety, emotion and behaviour regulation, and social stability. It is noteworthy that being within a CMHT may offer some containment and stability, even if this is not readily apparent. That said, there are potential negative effects of prolonged unfocused CMHT input, including ineffective resource use and the fostering of dependence as opposed to recovery. As noted already, clinicians should also be mindful of discharging in response to their countertransference.

### Limitations

There are limitations in applying the transtheoretical model to EUPD. It has typically been used in single health behaviours, e.g. smoking or alcohol misuse, whereas multiple complex behaviours are present in EUPD. We also note that a binary ready/not ready approach is an arbitrary and artificial oversimplification of what is a complex dynamic process, and it could be used inappropriately to obstruct access to treatment. However, our systematic search did not identify any evidence regarding increasing readiness in EUPD and, in the absence of other suggested frameworks, we believe our model is pragmatic and can aid clinical thinking and decision-making.

## Conclusion

EUPD is commonly encountered in mental health services, but some patients are not at a stage where they are ready to engage in specialist treatments. No guidelines exist regarding how to manage such patients, and prolonged unfocused treatment in CMHTs is not ideal.

We suggest that readiness for referral can be considered in terms of the transtheoretical model of stages of change. A range of approaches and non-specialist interventions exist which can enhance readiness and which can be used in a shared plan working towards referral for specialist treatment. Further research is required into which approaches may best increase readiness and what best practice is for patients who, despite intervention, remain unable to engage in specialist treatment.

## About the authors

**Matthew Roughley** is a specialty trainee at the Centre for Understanding Personality Disorder (CUSP), Deancross: Tower Hamlets Personality Disorder Service, Mile End Hospital, East London NHS Foundation Trust, UK. **Amy Maguire** is a counselling psychologist at the Centre for Understanding Personality Disorder (CUSP), Deancross: Tower Hamlets Personality Disorder Service, Mile End Hospital, East London NHS Foundation Trust, UK. **Grace Wood** is a People Participation team member at Trust Headquarters, East London NHS Foundation Trust, UK. **Tennyson Lee** is a consultant psychiatrist in psychotherapy at CUSP, Deancross: Tower Hamlets Personality Disorder Service, Mile End Hospital, East London NHS Foundation Trust, UK

## Supplementary material

For supplementary material accompanying this paper visit https://doi.org/10.1192/bjb.2020.48. click here to view supplementary material
